# Evaluating Global Inequality Using Decomposition Approach

**DOI:** 10.3389/fpsyg.2021.809670

**Published:** 2022-01-26

**Authors:** Ning Ma, Tsun Se Cheong, Jing Li

**Affiliations:** ^1^Hainan College of Economics and Business, Haikou, China; ^2^Department of Economics and Finance, Hang Seng University of Hong Kong, Siu Lek Yuen, Hong Kong SAR, China; ^3^Department of Geography and Resource Management, The Chinese University of Hong Kong, Shatin, Hong Kong SAR, China; ^4^Institute of Future Cities, The Chinese University of Hong Kong, Shatin, Hong Kong SAR, China

**Keywords:** inequality, global, decomposition analysis, Theil index, North–South divide

## Abstract

Given that there is no recent research on decomposition for global inequality, the aim of this study is to fill the gap in the literature by investigating global inequality with decomposition technique. The data of this study were compiled from the World Bank and decomposition by subgroups was conducted to evaluate the driving forces behind the evolution of inequality. Almost all the countries in the world were included in this study, and the study period spans from 2000 to 2017. The analysis was carried out in several stages to evaluate the issue of North–South divide, as well as the impacts of regional and income subgroups. There are several salient findings derived from this study. First, the results show that there was a gradual decline of international inequality within the study period. Second, there was still a large disparity between the developed and developing countries, and the inequality within the developing countries has aggravated further. Third, geographical location has exerted great impacts on global inequality and East Asia contributed about 40% to the overall decline in international inequality. Fourth, decline in inequality amongst the upper-middle-income countries also contributed substantially to the fall in international inequality. The results derived from this paper can provide pertinent information for the formulation of a comprehensive and coherent strategy in coordinating international efforts and managing inequality while promoting human development under the framework of the newly established Sustainable Development Goals.

## Introduction

Given that the Millennium Development Goals (MDGs) era has already come to a conclusion with the end of 2015, the General Assembly of the United Nations (UN) adopted the official document, *Transforming our world: the 2030 Agenda for Sustainable Development*, as the post-2015 global development agenda. This new universal agenda is made up of 17 new Sustainable Development Goals (SDGs) which are expected to stimulate global action over the next 15 years in areas of critical importance for humanity and the planet, namely, the economic, social and environmental dimensions of sustainable development. It is expected that the implementation of this ambitious agenda would be achieved with the participation of all countries, all stakeholders and all people (United Nations, 2015).

Although equality is one of the core values of the UN’ Millennium Declaration, the old MDGs only touch upon gender equality, and the targets of MDGs do not place enough emphasis on other forms of inequality (Melamed, 2012). However, with over 2 years of public consultation and engagement with civil society and other stakeholders around the world, the issue of inequality was finally integrated into the new agenda (United Nations, 2015) and was converted into the objective of the tenth goal, which is to “reduce inequality within and among countries.” It can be expected that the adoption of the new agenda by the United Nations will surely lead to a surge in the demand for policy research studies in international inequality. The changes in UN’s agenda call for a detailed research on the thorny issue of inequality for all the countries in the world so that policy implications can be drawn to assist countries in formulating inequality-alleviating policies.

Given the number of adverse impacts related to income inequality, a comprehensive study on inequality is justified to formulate policies that can alleviate inequality and ameliorate these adverse effects in the future. Country-specific regional disparity and income decomposition studies have been widely reported for developed (Karakoc, 2017; Kennedy et al., 2017; Kisiała and Suszyńska, 2017; Okabe and Kam, 2017; Bittencourt et al., 2019; Blanco and Ram, 2019) and developing (Choudhury and Chaterjee, 2016; Jang and Jeong, 2016; Paredes et al., 2016; Tian et al., 2016; Habibullah et al., 2017; Sehrawat and Giri, 2018; Calcagnini et al., 2019; Cevik and Correa-Caro, 2019; Le and Nguyen, 2019; Michálek and Výbošt́ok, 2019; Tchamyou et al., 2019) countries. However, to our knowledge there has been no recent study on the contributions of regional and income subgroups to international inequality for the world. This study aims to provide additional information concerning international income inequality for all the countries in the world so as to contribute to the literature in the post-MDGs era. This paper is divided into two parts. First, Theil-T and Theil-L indices are employed to provide an overview of the evolutionary patterns and trends of international inequality. Second, countries are divided into regional and income subgroups, and decomposition by subgroups is conducted to estimate the contributions of each of the subgroups and the inter-subgroup component to overall international inequality. The decomposition analysis can shed light on the underlying patterns of inequality, and quantify the level of the North–South divide. It can also reveal the relationship between inequality and different geographical and income subgroups, thereby pinpointing the crux of the problem of inequality.

The remainder of this paper is organized as follows. Section “Literature Review” reviews the relevant literature on international income inequality. Section “Methodology and Data” describes the methodology and data source. Section “Result and Discussion” conducts inequality measurement for all the countries and economies in the world; followed by computation of the contributions of different regional and income subgroups to overall inequality in an attempt to provide evidence on whether overall inequality can be mainly accounted for by the notorious North–South divide, or by the disparity within each of the different regional subgroup. Section “Conclusion” summarizes the research findings with policy implications.

## Literature Review

Many researchers claim that inequality has increased considerably with globalization (Krugman and Venables, 1995; Alderson and Nielsen, 2002; Ha, 2012; Ezcurra and Rodríguez-Pose, 2013). With the deepening of globalization, international inequality has reached record levels. In 2015, the top one percent of the global population owns half of all the world’s assets (Credit Suisse, 2015). This enormous level of disparity not only exerts damaging impacts on the progress of poverty reduction and economic growth, but also poses a threat to regional stability. Actually, the interlinkage and interdependence relationships created by increased globalization always carry the risk of contagion (Schmuckler, 2004). The countries are prone to social and economic instability in a globally interconnected economy as some scholar claimed that “the Southern predicament of instability and inequality does affect the economic and political well-being of the North itself” (Acharya, 1994).

It is well known that inequality can exert various adverse impacts on the progress of poverty reduction, the economic growth, and even social and political stability. Many researchers maintain that inequality exerts an adverse effect on poverty reduction (Rupasingha and Goetz, 2007; Zhuang, 2008; Fosu, 2009); while other studies claim that inequality has a negative impact on economic growth (Alesina and Rodrik, 1994; Persson and Tabellini, 1994; Alesina and Perotti, 1996; Deininger and Squire, 1998; Huang et al., 2009). Cheong and Wu (2015) found that inequality is positively correlated with the crime rate. Likewise, other researchers found that inequality can lead to different kinds of social dysfunction, such as mental illness, racism, social unrest and even political upheaval (Muller and Seligson, 1987; Alesina and Perotti, 1996; Wang and Hu, 1999; Acemoglu and Robinson, 2001; Wen, 2007; Dutt and Mitra, 2008; Wilkinson and Pickett, 2009; Knight, 2013).

Regarding the decomposition of international income inequality, there are typically three perspectives: North–South divide; income group; and regional effect. The global North–South divide has been studied for the service sector. Using a North–South growth model of endogenous industry location, it is found that trade integration leads to an increase in interregional real income inequality when the inter-sectoral knowledge spillovers from the manufacturing sectors are local (Fukuda, 2019). As for income group, Van Velthoven et al. (2019) used a panel data of many countries from 1975 to 2005 for analysis, and found that financial development, financial liberalization and banking crises are more influential than other factors in contributing to income redistribution. Based on a new panel data of Credit Suisse for 45 countries from 2000 to 2012, Islam and McGillivray (2019) found that the global wealth inequality is negatively associated with cross-country economic growth. Adopting Granger Causality Test and System Generalized Method of Moments Model of 158 countries and 86 middle income countries from 1960 to 2014, Vo et al. (2019) found causality from economic growth to income inequality and vice versa for middle income countries. Studying the datasets of household income from 67 European, American and Asian countries for a wide span of years, Tao et al. (2019) found income distribution for low and middle income class populations follows an exponential law. As for regional effect, Rapacki and Prochniak (2019) found that during 1995 to 2015, central and eastern European (the CEE or EU11) countries’ income levels converged to western European (the EU15) countries after obtaining the European Union memberships. Lee et al. (2019) applied the regression-based inequality decomposition approach to a panel data of 2006–2012, and found that financial development, urbanization, and globalization have a positive impact on income growth in China, yet only financial development has effect on promoting inclusive growth.

Although previous studies mentioned above provide important information on inequality, it is regrettable that there is no recent study focusing on international inequality and also the contributions of regional and income subgroups to global inequality. The aim of this study is to fill this gap in the literature by examining the relationship between inequality and these regional and income subgroups.

## Methodology and Data

Milanovic (2005) concludes that inequality can be measured from two different perspectives. The first approach is based on an unweighted measure so that it can only show the inequality amongst the countries without taking their population into consideration, whereas the second approach emphasizes the inequality of the people, and hence population is incorporated into the formula. The second approach is better as it takes the population of a country into consideration.

It is worth noting that many inequality measurements are available; however, the most common ones are the Gini coefficient and the Theil-T/Theil-L indices because they satisfy the property of income-zero-homogeneity and the Pigou-Dalton condition (Bourguignon, 1979). Without delving too much into the technicalities, the income-zero-homogeneity refers to the value of the inequality measurement, which remains unchanged when there is a scale change of the whole income distribution (Cheong, 2012), whilst the Pigou–Dalton principle suggests that a transfer of income from a rich person to a poor person should result in a decline in the inequality indicator, so long as the transfer does not reverse the ranking of the two in the income distribution (Cheong, 2012). It is worth noting that the Theil-T and Theil-L indices can be decomposed completely into the components of the subgroups (Bourguignon, 1979; Shorrocks, 1980, 1984). However, the Gini coefficient cannot satisfy the property of additive decomposability, and it cannot be decomposed completely into the components of subgroups (Yao, 1999). Therefore, the inequality measurement results based on the Theil index can be employed in the decomposition by subgroups in analyzing the relationship between inequality and regional and income subgroups. Specifically, the Theil-T and Theil-L indices (Theil, 1967, 1972) are employed in this study.

### Inequality Measurement

The formulae of the unweighted Theil-T and Theil-L, respectively, are:


(1) $$T\,h\,e\,i\,l-T=\sum_{i}\frac{Y_{i}}{Y}\,l\,n\,\frac{\frac{Y_{i}}{Y}}{\frac{1}{R}}$$

(2) $$T\,h\,e\,i\,l-L=\sum_{i}\frac{1}{R}\,l\,n\,\frac{\frac{1}{R}}{\frac{Y_{i}}{Y}}$$

where *R* is the total number of countries.

The formula of the population-weighted Theil-T is


(3) $$T\,h\,e\,i\,l-T=\sum_{i}\frac{Y_{i}}{Y}\,l\,n\,\frac{\frac{Y_{i}}{Y}}{\frac{n_{i}}{N}}$$

And the formula of the population-weighted Theil-L is:


(4) $$T\,h\,e\,i\,l-L=\sum_{i}\frac{n_{i}}{N}\,l\,n\,\frac{\frac{n_{i}}{N}}{\frac{Y_{i}}{Y}}$$

where *Y* is the total gross domestic product (GDP) of all the countries, *Y*_*i*_ is the GDP in country *i*, *N* is the total population in the world, and *n*_*i*_ is the population in country *i*.

### Decomposition by Subgroups

The decomposition of inequality by subgroups can be employed to determine the contributions of the subgroups to overall international inequality (For details, please refer to Theil, 1967, 1972). Overall inequality is then decomposed into the inequality existing between these subgroups (the inter-subgroup component) and the weighted sum of the inequalities existing within these subgroups (the intra-subgroup component) (Bourguignon, 1979; Shorrocks, 1980, 1984).

Overall inequality, *I*, can be decomposed into the sum of the intra-subgroup component and inter-subgroup component.


(5) $$I=\sum {\text{w}}_{\text{j}}\,I_{i\,n\,t\,r\,a,j}+I_{i\,n\,t\,e\,r}$$

where *W*_*j*_ is the weight for the *j*th regional subgroup, *I*_*intra,j*_ is the intra-subgroup inequality within regional subgroup *j*, and *I*_*inter*_ is the inter-subgroup component.

The weights of the Theil-T and Theil-L are not the same. Income weight should be used in Theil-T, whereas population weight should be used in Theil-L (Gustafsson and Li, 2002).


$$\mathrm{The}\mathrm{weight}\mathrm{of}\mathrm{Theil}-T\mathrm{for}\mathrm{regional}\mathrm{subgroup}j=\frac{{\text{Y}}_{\text{j}}}{\text{Y}}$$

(6) $$\mathrm{The}\,\mathrm{weight}\,\mathrm{of}\,\mathrm{Theil}-L\,\mathrm{for}\,\mathrm{regional}\,\mathrm{subgroup}\,j=\frac{{\text{n}}_{\text{j}}}{\text{N}}$$

where *Y* is the world GDP, *Y*_*j*_ is the regional GDP in subgroup *j*, *N* is the world population, *n*_*i*_ is the population in subgroup *j*. So *W*_*j*_, the weight for the *j*th subgroup, is $\frac{{\text{Y}}_{\text{j}}}{\text{Y}}$ for Theil-T and $\frac{{\text{n}}_{\text{j}}}{\text{N}}$ for Theil-L where *Y*_*j*_ is the regional GDP in subgroup *j* and *n*_*j*_ is the population. The full dataset will be divided according to different schemes of subgroup, so the impacts of these subgroups can be observed in details.

### Data

The data of all the countries employed in this study were compiled from the World Bank^1^. For each country, the data of GDP and population were collected for the computation of the Theil-L and Theil-T indices. It is worth noting that the same countries should be employed for the measurement of inequality for each year in the study period. The omission or addition of a country in a year may result in sudden change of the inequality measurement in that particular year, thereby providing misleading information on the evolution of inequality. Therefore, the countries employed in this study are the same across time. Almost all the countries listed in the World Bank World Development Indicators Database are included in this study, however, a few countries are excluded because of data unavailability. The study period spans from 2000 to 2017 for a total of 18 years.

There are several stages in this study. In the first stage, all the countries in the database were used to compute the Theil-L and Theil-T indices for the world. Then, in the second stage, the data were divided into two smaller data sets, namely, the North and South subgroups. The data were then divided into seven regional subgroups according to the regional classification proposed by the World Bank, namely, the East Asia and Pacific, Europe and Central Asia, Latin America and Caribbean, Middle East and North Africa, North America, South Asia, and Sub-Saharan Africa in the third stage of the study. In the fourth stage, the countries are further classified into 17 regional subgroups so as to provide a detailed analysis on the relationship between international inequality and regional subgroups. Finally, in the fifth stage of this study, the data were separated into four income groups as defined by the World Bank, namely, low, lower-middle, upper-middle, and high income groups. This classification allows one to evaluate the inequality amongst these income groups and within them in great detail.

## Results and Discussion

The first analysis is based on the complete dataset. Section “North–South Divide” comprises the North (developed countries) dataset and the South (developing countries) dataset for analyses. Section “Regional Effect” provides a comparison of all datasets by regions and sub-regions, while Section “Income Group” provide the findings derived from the analyses which are based on income groups.

**FIGURE 1:**
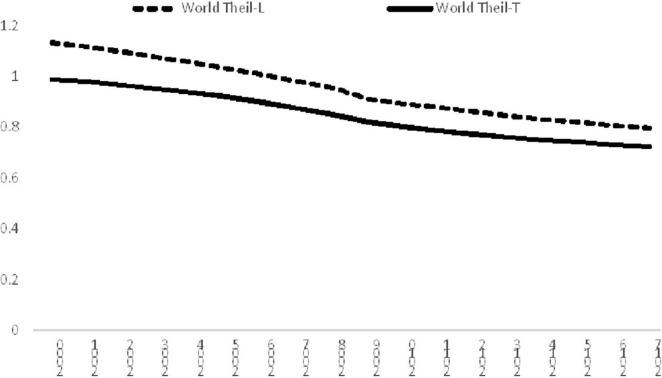
Income inequality in the World, 2000–2017. Source: Authors’ calculation.

Figure 1 shows the income inequality of the world from 2000 to 2017. It can be observed that there was a gradual decline of international inequality within the study period. The Theil-L index dropped from 1.13 in 2000 to 0.80 in 2017, while the Theil-T index declined from 0.99 to 0.72 in that period. Although the values of the indices differ, both indices indicated that there was a 27–30 percent drop in international inequality. This is a very encouraging finding for pursuing the SDGs as it shows that the disparity amongst the countries has decreased across time.

### North–South Divide

**FIGURE 2:**
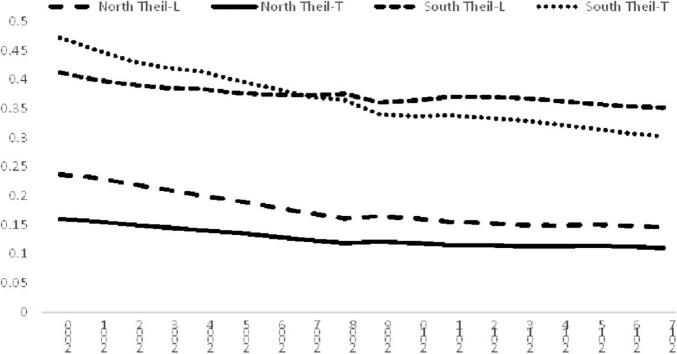
Income inequality for the North and South, 2000–2017. Source: Authors’ calculation.

The evolution of inequalities within the North and South is shown in Figure 2. There are two salient findings: First, the inequalities within these two regions both declined steadily in the study period. However, it can be observed that the decline of the Theil-L index in the South is much slower than the Theil-T index in that region, thereby creating an intersection for the two indices in 2007.

Another interesting finding is that the inequality in the South was much higher than the North. Using Theil-L in inequality measurement, the ratio of the inequality of the South to the North was 1.74 in 2000, it then increased to 2.42 in 2017. However, the ratio of the inequality of the South to the North based on Theil-T index was 2.95 in the beginning of the study period, and it remained roughly the same, resulting in a ratio of 2.74 in 2017. The findings suggest that inequality in the South is about 2.5 times higher than that in the North in 2017. This finding is alarming as it highlights the fact that the inequality amongst developing countries is significantly higher than the inequality amongst developed countries.

**FIGURE 3:**
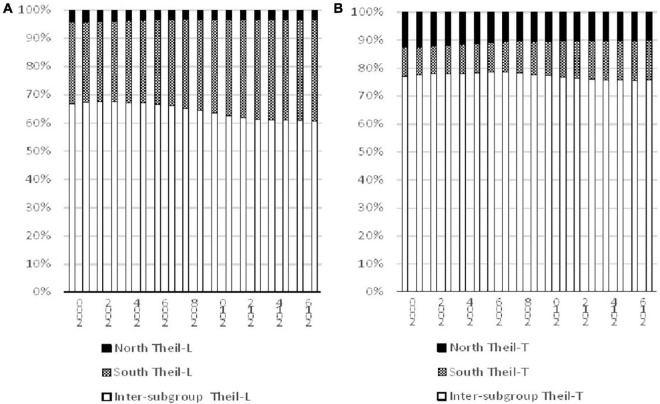
Decomposition of inequality for the North and South, 2000–2017. Source: Authors’ calculation.

The decomposition results of the North and South are shown in Figure 3, while Figure 3A is based on Theil-L index and Figure 3B is based on Theil-T index. Similar conclusions can be reached even though the contribution percentage of the two measurements are not exactly the same. It is noteworthy that the largest contributor to global inequality is found to be the inter-subgroup component, followed by the inequality within the South and then the disparity within the North. The inter-subgroup component has declined steadily across time within the study period, however, it was still the largest contributor in 2017. The decline in percentage contribution was replaced by the rise in the inequality within the South. This finding is distributing as it indicates that although there was a decline of overall inequality in the world, there was still a large disparity between the developed and developing countries, and the inequality within the developing countries has aggravated further.

Figure 1 shows that there was a decline of inequality within the study period, however, Figure 3 indicates that the inequality within the South had increased in percentage contribution, therefore, it is difficult to evaluate the actual impacts of the South by the two figures. Therefore, following the practice employed by Cheong and Wu (2012), a decomposition of change is implemented to examine the contribution to the change in inequality within this period. By using decomposition on the change of inequality rather than the inequality index itself, the underlying determinant behind the change can be revealed in detail. The findings are shown in Table 1. It shows that the largest contributor of the change in overall inequality is the inter-subgroup component, thereby suggesting that the decline in the disparity between the North and South contributed to more than 80% of the overall decline in international inequality. For Theil-T index, the decline in the inequality within the North contributed about 20% to the overall decline, while it is 6.7% for the Theil-L index. However, the contribution of the South is quite mixed, it is 11.7% for Theil-L, but 0.38% for Theil-T which suggests that the inequality within the South had aggravated overall inequality further.

**TABLE 1:** Decomposition of change in inequality for the North and South, 2000–2017.

	2000	2000	2017	2017	Nominal change		Change (%)	
	Theil-T	Theil-L	Theil-T	Theil-L	Theil-T	Theil-L	Theil-T	Theil-L
World	0.99	1.13	0.72	0.80	−0.27	−0.34	−100.00	−100.00
Global North	0.13	0.05	0.07	0.03	−0.05	−0.02	−19.82	−6.73
Global South	0.10	0.33	0.10	0.29	0.00	−0.04	0.38	−11.67
Inter-subgroup	0.76	0.76	0.55	0.48	−0.21	−0.27	−80.56	−81.60

*Source: Authors’ calculation.*

### Regional Effect

In order to examine the impacts of regional subgroups in detail, the full dataset was divided into seven smaller regional datasets according to the regional classification proposed by the World Bank for assessing the trend and contribution of these regional subgroups. Table 2 shows the inequality indices for these regions from 2000 to 2017, and it can be observed that the inequalities within most of the regions declined in that period, except for the region of South Asia. The Theil-L index of South Asia increased for 86%, while the Theil-T index increased for 62%. It is worth mentioning that there are two regional subgroups which had a significant drop in inequality, namely, East Asia and Pacific and Europe and Central Asia. The findings derived from the two indices are very similar: the inequality within East Asia and Pacific declined for about 60%, while that within Europe and Central Asia declined for about 30%. In the beginning of the study period, the East Asia and Pacific region had the highest level of inequality in the world, however, the disparity had been mitigated considerably and its Theil-L index was lower than many regions in 2017. The reduction in inequality within this region is phenomenal, and its impact on overall inequality will be investigated further by using decomposition techniques.

**TABLE 2:** Income inequality for seven regional subgroups, 2000–2017.

	East Asia and Pacific	Europe and Central Asia	Latin America and Caribbean	Middle East and North Africa	North America	South Asia	Sub-Saharan Africa
	**Theil-L**						

2000	0.80	0.54	0.13	0.43	0.00	0.02	0.50
2001	0.77	0.53	0.13	0.41	0.00	0.02	0.50
2002	0.73	0.51	0.13	0.40	0.00	0.02	0.51
2003	0.70	0.49	0.13	0.43	0.00	0.02	0.51
2004	0.67	0.47	0.13	0.42	0.00	0.02	0.50
2005	0.63	0.45	0.13	0.41	0.00	0.02	0.50
2006	0.59	0.43	0.12	0.41	0.00	0.02	0.50
2007	0.55	0.41	0.12	0.40	0.00	0.02	0.50
2008	0.52	0.40	0.12	0.39	0.00	0.02	0.49
2009	0.47	0.40	0.12	0.36	0.00	0.02	0.48
2010	0.45	0.39	0.12	0.35	0.00	0.02	0.47
2011	0.43	0.39	0.12	0.38	0.00	0.02	0.46
2012	0.41	0.38	0.12	0.38	0.00	0.03	0.45
2013	0.39	0.37	0.12	0.38	0.00	0.03	0.44
2014	0.38	0.37	0.12	0.39	0.00	0.03	0.43
2015	0.36	0.38	0.11	0.40	0.00	0.03	0.42
2016	0.35	0.38	0.11	0.41	0.00	0.03	0.41
2017	0.34	0.37	0.11	0.41	0.00	0.03	0.39

	**Theil-T**						

2000	1.03	0.37	0.10	0.55	0.00	0.02	0.52
2001	0.99	0.36	0.10	0.53	0.00	0.02	0.51
2002	0.95	0.35	0.10	0.51	0.00	0.02	0.51
2003	0.91	0.34	0.10	0.53	0.00	0.02	0.50
2004	0.88	0.32	0.10	0.52	0.00	0.02	0.50
2005	0.83	0.31	0.10	0.51	0.00	0.02	0.50
2006	0.78	0.30	0.09	0.49	0.00	0.02	0.50
2007	0.73	0.29	0.09	0.47	0.00	0.02	0.50
2008	0.68	0.28	0.09	0.45	0.00	0.02	0.49
2009	0.62	0.28	0.09	0.41	0.00	0.02	0.47
2010	0.59	0.28	0.09	0.40	0.00	0.02	0.46
2011	0.55	0.28	0.09	0.42	0.00	0.02	0.46
2012	0.53	0.27	0.09	0.43	0.00	0.03	0.45
2013	0.50	0.27	0.09	0.43	0.00	0.03	0.44
2014	0.48	0.27	0.08	0.44	0.00	0.03	0.43
2015	0.46	0.27	0.08	0.45	0.00	0.03	0.42
2016	0.44	0.27	0.08	0.44	0.00	0.03	0.41
2017	0.42	0.27	0.08	0.43	0.00	0.03	0.40

*Source: Authors’ calculation.*

The results of decomposition of inequality by the seven regional subgroups are shown in Table 3. It shows that the contribution of the inter-subgroup component had increased from 2000 to 2017, thereby indicating that the disparity amongst the regions had gained in relative importance across time. Another interesting finding is that the contributions of East Asia and Pacific and Europe and Central Asia had both decreased, while the contributions of other regions had increased. The contribution of East Asia and Pacific had decreased from 24 to 13% for Theil-L and 23–17% for Theil-T, while the contribution of Europe and Central Asia had dropped for a small amount. However, it is worth mentioning that although the region of East Asia and Pacific had declined significantly, it remained to be the largest contributor to world inequality in 2017.

**TABLE 3:** Decomposition of inequality for seven regional subgroups, 2000–2017.

	Theil-L							
**Percent contribution**	**East Asia and Pacific**	**Europe and Central Asia**	**Latin America and Caribbean**	**Middle East and North Africa**	**North America**	**South Asia**	**Sub-Saharan Africa**	**Inter-subgroup**

2000	23.67	6.68	0.96	1.88	0.00	0.36	4.54	61.91
2001	22.92	6.56	0.98	1.86	0.00	0.34	4.63	62.71
2002	22.14	6.37	0.99	1.84	0.00	0.36	4.87	63.44
2003	21.42	6.15	0.99	2.00	0.00	0.39	5.07	63.98
2004	20.83	5.92	1.04	2.02	0.00	0.42	5.19	64.58
2005	20.05	5.77	1.04	2.06	0.00	0.44	5.36	65.27
2006	19.16	5.62	1.05	2.09	0.00	0.48	5.56	66.03
2007	18.25	5.50	1.07	2.10	0.00	0.51	5.80	66.77
2008	17.40	5.38	1.09	2.12	0.00	0.51	5.93	67.56
2009	16.55	5.66	1.12	2.09	0.00	0.57	6.16	67.85
2010	16.17	5.64	1.19	2.12	0.00	0.66	6.28	67.96
2011	15.43	5.56	1.20	2.30	0.00	0.70	6.37	68.45
2012	14.98	5.49	1.20	2.40	0.00	0.74	6.46	68.73
2013	14.64	5.42	1.21	2.49	0.00	0.77	6.57	68.90
2014	14.20	5.49	1.19	2.60	0.00	0.82	6.62	69.08
2015	13.77	5.64	1.15	2.76	0.00	0.89	6.63	69.17
2016	13.37	5.68	1.12	2.87	0.00	0.96	6.59	69.41
2017	13.03	5.66	1.12	2.91	0.00	0.98	6.57	69.73

	**Theil-T**							

	**East Asia and Pacific**	**Europe and Central Asia**	**Latin America and Caribbean**	**Middle East and North Africa**	**North America**	**South Asia**	**Sub-Saharan Africa**	**Inter-subgroup**

2000	22.55	12.96	0.74	1.84	0.00	0.04	0.80	61.07
2001	22.13	12.92	0.74	1.78	0.00	0.04	0.82	61.57
2002	21.78	12.62	0.75	1.73	0.00	0.04	0.86	62.23
2003	21.48	12.18	0.74	1.86	0.00	0.05	0.88	62.82
2004	21.19	11.79	0.77	1.90	0.00	0.05	0.90	63.39
2005	20.76	11.47	0.76	1.91	0.00	0.05	0.94	64.10
2006	20.21	11.27	0.76	1.92	0.00	0.06	0.98	64.79
2007	19.78	11.14	0.77	1.88	0.00	0.07	1.04	65.33
2008	19.31	10.99	0.80	1.90	0.00	0.07	1.08	65.85
2009	18.75	11.24	0.82	1.85	0.00	0.08	1.13	66.14
2010	18.79	11.14	0.87	1.85	0.00	0.09	1.15	66.10
2011	18.20	11.07	0.88	1.99	0.00	0.10	1.18	66.57
2012	18.05	10.83	0.88	2.08	0.00	0.11	1.20	66.85
2013	17.97	10.62	0.89	2.15	0.00	0.12	1.23	67.02
2014	17.52	10.66	0.86	2.22	0.00	0.13	1.24	67.37
2015	17.12	10.82	0.80	2.30	0.00	0.14	1.22	67.60
2016	16.81	10.89	0.76	2.34	0.00	0.16	1.19	67.85
2017	16.56	10.85	0.75	2.30	0.00	0.17	1.17	68.21

*Source: Authors’ calculation.*

Given that the fall in inequality in the East Asia and Pacific region is so huge, decomposition of change in inequality was conducted to evaluate the contribution of all regional subgroups to the change in overall inequality. Table 4 shows the results of the decomposition and it can be observed that the inter-subgroup component contributed more than 40% for the overall decline in inequality, while the contribution of the inequality within East Asia and Pacific region was 39% for Theil-T and 49% for Theil-L. This is an important finding as it shows that almost 50% (as measured by Theil-L index) of the drop in overall global inequality can be attributed to the fall in inequality within East Asia and Pacific. The decline in inequality within East Asia and Pacific not only mitigated disparity within this region, but also help reduce global inequality significantly. For the other regions, it can be observed that the contribution of Europe and Central Asia to the decline in global inequality was 19% for Theil-T and 9% for Theil-L, while the contribution of the other five regions were negligible.

**TABLE 4:** Decomposition of change in inequality for seven regional subgroups, 2000–2017.

	2000	2000	2017	2017	Nominal change		Change (%)	
	Theil-T	Theil-L	Theil-T	Theil-L	Theil-T	Theil-L	Theil-T	Theil-L
World	0.99	1.13	0.72	0.80	−0.27	−0.34	−100.00	−100.00
East Asia and Pacific	0.22	0.27	0.12	0.10	−0.10	−0.16	−38.90	−48.87
Europe and Central Asia	0.13	0.08	0.08	0.05	−0.05	−0.03	−18.71	−9.08
Latin America and Caribbean	0.01	0.01	0.01	0.01	0.00	0.00	−0.72	−0.59
Middle East and North Africa	0.02	0.02	0.02	0.02	0.00	0.00	−0.58	0.55
North America	0.00	0.00	0.00	0.00	0.00	0.00	0.00	0.00
South Asia	0.00	0.00	0.00	0.01	0.00	0.00	0.30	1.13
Sub-Saharan Africa	0.01	0.05	0.01	0.05	0.00	0.00	0.20	0.26
Inter-subgroup	0.60	0.70	0.49	0.56	−0.11	−0.15	−41.61	−43.39

*Source: Authors’ calculation.*

Although the results derived from the decomposition by the seven regional subgroups suggests that the region of East Asia and Pacific and the region of Europe and Central Asia contributed significantly to the decline of inequality within the study period; however, the regional classification proposed by the World Bank is too broad and thus it cannot provide a clear picture to the regional impacts. Therefore, the dataset was further separately into 17 regions so that the analysis could be conducted in detail. However, in order to save space, only the results of Theil-T index will be provided, but it is worth mentioning that the results derived from the two indices are very similar. Interested readers may contact the authors for further details.

Table 5 shows the evolution of inequalities within the 17 regions. It is found that the inequalities in three regions had increased, namely, Central Asia, South Asia, and West Africa, while the inequalities in other regions had decreased. One important fact can be deduced for Central Asia, by comparing with the finding derived from Table 3 which shows that the inequality in Europe and Central Asia had decreased, it implies that the drop in inequality within that region can be attributed to the decline in inequality within the regions of EU and Other EU, rather than Central Asia. Turning to another important region as shown in Table 3, namely, East Asia and Pacific, it can be observed that the Theil-T index within Pacific region had decreased from 0.04 to 0.03 in that period, while the Theil-T index of East Asia had declined from 1.04 to 0.33.

**TABLE 5:** Income inequality (Theil-T) for 17 regional subgroups, 2000–2017.

	Theil-T								
	**Caribbean**	**Central Africa**	**Central Asia**	**East Africa**	**East Asia**	**EU28**	**Middle East**	**North Africa**	**North America**

2000	0.52	0.64	0.39	0.33	1.04	0.09	0.48	0.06	0.00
2001	0.54	0.65	0.43	0.32	1.00	0.08	0.47	0.06	0.00
2002	0.54	0.63	0.45	0.33	0.95	0.08	0.46	0.06	0.00
2003	0.54	0.61	0.47	0.35	0.90	0.07	0.49	0.06	0.00
2004	0.57	0.57	0.48	0.33	0.86	0.07	0.46	0.06	0.00
2005	0.53	0.56	0.49	0.32	0.81	0.07	0.45	0.07	0.00
2006	0.50	0.53	0.51	0.32	0.75	0.07	0.43	0.06	0.00
2007	0.48	0.53	0.51	0.33	0.68	0.06	0.40	0.06	0.00
2008	0.46	0.50	0.49	0.32	0.63	0.06	0.39	0.05	0.00
2009	0.45	0.49	0.47	0.31	0.55	0.06	0.36	0.05	0.00
2010	0.45	0.49	0.47	0.30	0.52	0.06	0.34	0.04	0.00
2011	0.44	0.49	0.48	0.28	0.48	0.06	0.36	0.04	0.00
2012	0.44	0.49	0.47	0.26	0.45	0.06	0.37	0.05	0.00
2013	0.43	0.47	0.46	0.26	0.42	0.06	0.38	0.05	0.00
2014	0.42	0.46	0.46	0.24	0.39	0.06	0.39	0.05	0.00
2015	0.42	0.45	0.44	0.24	0.37	0.06	0.41	0.05	0.00
2016	0.41	0.45	0.42	0.23	0.35	0.06	0.39	0.05	0.00
2017	0.40	0.44	0.42	0.23	0.33	0.06	0.39	0.05	0.00

	**Central America**	**Other EU**	**Pacific**	**South Africa**	**South America**	**South Asia**	**Southeast Asia**	**West Africa**	

2000	0.10	0.57	0.04	0.29	0.06	0.02	0.37	0.08	
2001	0.10	0.56	0.04	0.29	0.06	0.02	0.35	0.08	
2002	0.10	0.53	0.04	0.29	0.06	0.02	0.35	0.09	
2003	0.09	0.50	0.04	0.29	0.06	0.02	0.35	0.09	
2004	0.10	0.47	0.04	0.29	0.06	0.02	0.35	0.10	
2005	0.09	0.45	0.04	0.28	0.06	0.02	0.35	0.11	
2006	0.09	0.42	0.04	0.28	0.05	0.02	0.36	0.11	
2007	0.09	0.40	0.04	0.27	0.05	0.02	0.36	0.11	
2008	0.09	0.38	0.04	0.27	0.05	0.02	0.34	0.12	
2009	0.09	0.40	0.04	0.26	0.05	0.02	0.33	0.13	
2010	0.09	0.39	0.04	0.26	0.05	0.02	0.34	0.13	
2011	0.09	0.37	0.04	0.26	0.05	0.02	0.35	0.13	
2012	0.09	0.36	0.04	0.25	0.05	0.03	0.34	0.13	
2013	0.09	0.35	0.04	0.25	0.05	0.03	0.33	0.13	
2014	0.09	0.35	0.03	0.25	0.05	0.03	0.33	0.13	
2015	0.09	0.36	0.03	0.25	0.04	0.03	0.32	0.13	
2016	0.09	0.36	0.03	0.25	0.04	0.03	0.31	0.12	
2017	0.09	0.35	0.03	0.26	0.04	0.03	0.31	0.11	

*Source: Authors’ calculation.*

The decomposition results are shown in Table 6. There are several salient findings: First, the contribution of the inter-subgroup was 72.7% in 2000 and it increased to 79.9% in 2017. It shows that the disparity amongst the regions was huge, implying that geographical location had played a major role in global inequality. Second, based on Table 6, in 2000, the second largest contributor was the inequality within East Asia (18.2%), followed by Other EU (3.0%) and EU28 (2.6%). However, in 2017, the contribution of East Asia declined to the value of 10.4%, while the contribution of Other EU was 2.8%, and EU28 was 1.9%. It is worth noting that these three regions were still the largest contributors in 2017, however, their significance had decreased steadily in the study period.

**TABLE 6:** Decomposition of inequality (Theil-T) for 17 regional subgroups, 2000–2017.

	2000	2001	2002	2003	2004	2005	2006	2007	2008	2009	2010	2011	2012	2013	2014	2015	2016	2017
Caribbean	0.2	0.2	0.2	0.2	0.3	0.3	0.2	0.2	0.2	0.2	0.2	0.2	0.2	0.2	0.2	0.2	0.2	0.2
Central Africa	0.1	0.1	0.1	0.1	0.1	0.1	0.1	0.1	0.1	0.1	0.1	0.1	0.1	0.1	0.1	0.1	0.1	0.1
Central Asia	0.1	0.1	0.1	0.1	0.1	0.1	0.2	0.2	0.2	0.2	0.2	0.2	0.2	0.2	0.2	0.2	0.2	0.2
East Africa	0.1	0.1	0.1	0.1	0.1	0.1	0.1	0.1	0.1	0.1	0.1	0.1	0.1	0.1	0.1	0.1	0.1	0.1
East Asia	18.2	17.8	17.3	16.9	16.6	16.1	15.4	14.8	14.2	13.4	13.3	12.6	12.3	12.1	11.5	11.1	10.7	10.4
EU28	2.6	2.5	2.4	2.3	2.2	2.1	2.1	2.0	1.9	2.0	2.0	2.1	2.1	2.1	2.0	2.0	2.0	1.9
Middle East	1.3	1.2	1.2	1.3	1.3	1.3	1.3	1.3	1.3	1.3	1.2	1.3	1.4	1.5	1.5	1.6	1.6	1.6
North Africa	0.0	0.0	0.0	0.0	0.1	0.1	0.0	0.0	0.0	0.0	0.0	0.0	0.0	0.1	0.1	0.1	0.1	0.1
North America	0.0	0.0	0.0	0.0	0.0	0.0	0.0	0.0	0.0	0.0	0.0	0.0	0.0	0.0	0.0	0.0	0.0	0.0
Central America	0.2	0.2	0.2	0.2	0.2	0.2	0.2	0.2	0.2	0.2	0.2	0.2	0.2	0.2	0.2	0.2	0.2	0.2
Other EU	3.0	3.0	2.9	2.8	2.7	2.7	2.7	2.7	2.7	2.8	2.8	2.7	2.7	2.7	2.8	2.8	2.8	2.8
Pacific	0.1	0.1	0.1	0.1	0.1	0.1	0.1	0.1	0.1	0.1	0.1	0.1	0.1	0.1	0.1	0.1	0.1	0.1
South Africa	0.2	0.2	0.2	0.2	0.2	0.2	0.2	0.2	0.3	0.3	0.3	0.3	0.3	0.3	0.3	0.3	0.3	0.3
South America	0.3	0.3	0.3	0.3	0.3	0.3	0.3	0.3	0.3	0.3	0.4	0.4	0.4	0.4	0.3	0.3	0.3	0.3
South Asia	0.0	0.0	0.0	0.0	0.1	0.1	0.1	0.1	0.1	0.1	0.1	0.1	0.1	0.1	0.1	0.1	0.2	0.2
Southeast Asia	0.9	0.9	0.9	0.9	1.0	1.0	1.1	1.1	1.1	1.2	1.3	1.4	1.4	1.4	1.5	1.5	1.5	1.5
West Africa	0.0	0.0	0.1	0.1	0.1	0.1	0.1	0.1	0.1	0.1	0.1	0.1	0.1	0.1	0.2	0.2	0.1	0.1
Inter-subgroup	72.7	73.3	73.9	74.3	74.7	75.2	75.9	76.4	77.1	77.7	77.6	78.1	78.2	78.3	78.8	79.1	79.6	79.9

*Source: Authors’ calculation.*

In order to reveal the contribution to the change in inequality in greater detail, the decomposition of change of inequality was conducted, and the results are shown in Table 7. The inter-subgroup component contributed more than 50% to the overall decline in global inequality. Surprisingly, East Asia also contributed about 40% to this decline, thereby implying that an extremely large portion of reduction in global inequality can be attributed to inequality alleviation in East Asia alone. The mitigation of disparity within East Asia not only alleviated the inequality within this region but also led to 40% of the drop in global inequality. This fact can be explained by the huge population living in East Asia and the rapid increase in income in this region within the study period. Turning to other regions, the EU28 subgroup contributed 4.3% for Theil-T and 1.3% for Theil-L, while Other EU subgroup contributed 3.7% for Theil-T and 2.7% for Theil-L.

**TABLE 7:** Decomposition of change in inequality for 17 regional subgroups, 2000–2017.

	2000	2000	2017	2017	Nominal change		Change (%)	
	Theil-T	Theil-L	Theil-T	Theil-L	Theil-T	Theil-L	Theil-T	Theil-L
World	0.99	1.13	0.72	0.80	−0.27	−0.34	−100.00	−100.00
Caribbean	0.00	0.00	0.00	0.00	0.00	0.00	−0.26	−0.08
Central Africa	0.00	0.01	0.00	0.01	0.00	0.00	−0.03	0.03
Central Asia	0.00	0.00	0.00	0.00	0.00	0.00	0.28	0.13
East Africa	0.00	0.01	0.00	0.01	0.00	0.00	0.04	−0.35
East Asia	0.18	0.21	0.08	0.05	−0.10	−0.15	−39.52	−45.75
EU28	0.03	0.01	0.01	0.00	−0.01	−0.01	−4.31	−1.53
Middle East	0.01	0.01	0.01	0.01	0.00	0.00	−0.37	0.91
North Africa	0.00	0.00	0.00	0.00	0.00	0.00	0.00	−0.04
North America	0.00	0.00	0.00	0.00	0.00	0.00	0.00	0.00
Central America	0.00	0.00	0.00	0.00	0.00	0.00	−0.15	−0.11
Other EU	0.03	0.02	0.02	0.01	−0.01	−0.01	−3.67	−2.67
Pacific	0.00	0.00	0.00	0.00	0.00	0.00	−0.03	−0.01
South Africa	0.00	0.01	0.00	0.01	0.00	0.00	0.00	−0.15
South America	0.00	0.00	0.00	0.00	0.00	0.00	−0.34	−0.44
South Asia	0.00	0.00	0.00	0.01	0.00	0.00	0.30	1.13
Southeast Asia	0.01	0.02	0.01	0.02	0.00	0.00	0.76	−1.22
West Africa	0.00	0.00	0.00	0.01	0.00	0.00	0.21	1.11
Inter-subgroup	0.72	0.81	0.58	0.64	−0.14	−0.17	−52.93	−50.98

*Source: Authors’ calculation.*

### Income Group

**FIGURE 4:**
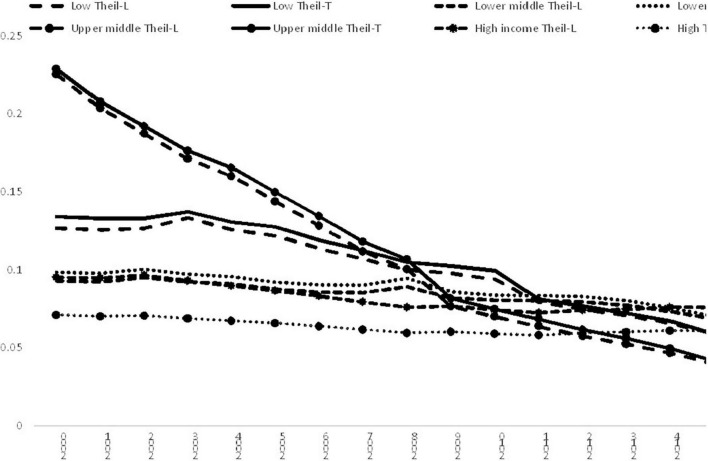
Income inequality for different income subgroups, 2000–2017. Source: Authors’ calculation.

We have investigated the North–South divide, and the impacts of regional subgroup, now we analyses the impacts of income so as to offer a comprehensive analysis. The full dataset was separated into four smaller datasets based on classification of income as defined by the World Bank. The trend and evolution of inequalities within these income subgroups are shown in Figure 4. It can be observed that the inequalities within all the income subgroups had declined in the study period. In 2017, the Upper-middle-income subgroup had the lowest level of inequality, followed by the Low-income group. However, conclusions on the other income subgroups differ according to the Theil index used in calculation though the difference is very small. For Theil-L, High-income subgroup had the highest level of inequality, followed by lower-middle-income countries; while the Theil-T values for these two regions were nearly the same in 2017.

**FIGURE 5:**
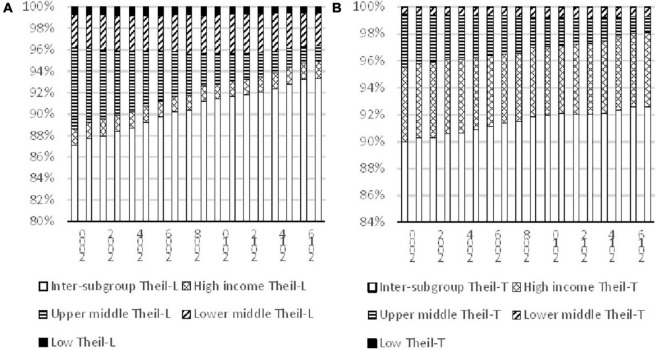
Decomposition of inequality for different income subgroups, 2000–2017. Source: Authors’ calculation.

The results of decomposition by income subgroups are shown in Figure 5. Figure 5A is based on Theil-L index and Figure 5B is based on Theil-T index. It can be observed that both indices indicate that the contribution of the inter-subgroup had increased from 2000 to 2017, along with a decline in contribution of the inequality within the Upper-middle-income subgroup. The contribution of the inter-subgroup as measured by Theil-L index increased from 87% in 2000 to 93% in 2017, while the measurement based on Theil-T index changed from 90 to 93%. For Theil-T index in 2017, the second highest contributor to overall inequality is the High-income subgroup, followed by the Upper-middle-income subgroups, then the Lower-middle-income subgroups, while the Low-income subgroup contributed the least. However, the measurement based on Theil-L is a bit different and the second largest contributor is the Lower-middle-income subgroups though the ordering of the other subgroups were the same as those of Theil-T.

Table 8 shows the results of decomposition for the different income subgroups. It can be observed that the contribution of the inter-subgroup was 72% for Theil-L and 83% for Theil-T. The second largest contributor was the Upper-middle-income subgroup and its contribution was 11% for Theil-T and 22% for Theil-L. The total contribution of the inter-subgroup component and the Upper-middle-income subgroup was more than 94% no matter which index was employed in calculation. It is evident that the Upper-middle-income subgroup played a major role in global inequality and affected the evolution of disparity in the world considerably.

**TABLE 8:** Decomposition of change in inequality for different income subgroups, 2000–2017.

	2000	2000	2017	2017	Nominal change		Change (%)	
	Theil-T	Theil-L	Theil-T	Theil-L	Theil-T	Theil-L	Theil-T	Theil-L
World	0.99	1.13	0.72	0.80	−0.27	−0.34	−100.00	−100.00
Low income	0.00	0.01	0.00	0.00	0.00	0.00	−0.06	−0.95
Low middle income	0.00	0.04	0.01	0.02	0.00	−0.01	0.03	−3.18
Upper middle income	0.04	0.09	0.01	0.01	−0.03	−0.07	−11.26	−22.12
High income	0.06	0.02	0.04	0.01	−0.02	0.00	−5.84	−1.32
Inter-subgroup	0.89	0.99	0.67	0.74	−0.22	−0.24	−82.87	−72.43

*Source: Authors’ calculation.*

In summary, there are two major findings derived from the analyses. First, referring to the analysis based on decomposition by regional subgroup, except for the inter-subgroup component, the most important contributor was identified to be the East Asia region. Second, the results derived from decomposition by income subgroups shows that, except for the inter-subgroup component, the Upper-middle-income subgroup was the largest contributor. By combining the two findings, it is of interest to investigate if there is any country which belongs to both the subgroups of East Asia and Upper-middle-income at the same time. It is found that there is only one country which fits both classification, and it is identified to be China. It pinpoints the importance of China in global inequality alleviation from 2000 to 2017. Economic development in China not only reduce disparity within East Asia and ameliorate inequality amongst the upper-middle-income countries, but also mitigate global inequality to a large extent.

## Conclusion

The United Nations adopted the new Sustainable Development Goals (SDGs) and formulated a new global development agenda for all the countries in 2015. Given that the aim of the tenth goal of the SDGs is to reduce inequality within and among countries, therefore, it is of interest to investigate global inequality and its evolution across time. Inequality decomposition is a valuable tool for this strand of research as it can reveal the contribution of each component to global inequality in great detail. However, it is worth noting that there is no recent research on global international inequality and its decomposition, therefore the objective of this study is to fill the gap in the literature by investigating global inequality with decomposition technique.

The data of this study were compiled from the World Bank and decomposition by subgroups was conducted to evaluate the driving forces behind the evolution of inequality. Almost all the countries in the world were included in this study, and the study period spans from 2000 to 2017. The analysis was carried out in several stages to evaluate the issue of North–South divide, as well as the impacts of regional and income subgroups.

There are several salient findings derived from this study. The results show that there was a gradual decline of international inequality within the study period. This seems to be an encouraging finding, however, the decomposition results reveal that there are many worrisome issues behind the fall in international inequality. For the North–South divide, it is found that the inequality in the South was 2.5 times higher than that in the North in 2017. Another interesting finding is that the inequalities within these two regions both declined steadily in the study period. However, there was still a large disparity between the developed and developing countries, and the inequality within the developing countries has aggravated further.

Turning to the impacts of regional subgroups, three regions had increased in inequality, namely, Central Asia, South Asia, and West Africa, while the inequalities in other regions had decreased. The disparity amongst the regions was huge, implying that geographical location had played a major role in global inequality. Although the contributions of the regions of East Asia, Other EU, and EU28 had declined significantly, they were still the largest contributor in 2017. East Asia contributed about 40% to the overall decline in international inequality.

The analysis based on income subgroups shows that the total contribution of the inter-subgroup component and the Upper-middle-income subgroup was more than 94%, thereby indicating that the Upper-middle-income subgroup played a major role in global inequality. It is notable that China is the only country which fits both classification of East Asia and Upper-middle-income. Therefore, the findings pinpoint the importance of China in global inequality alleviation. It is fair to comment that economic development in China not only reduced disparity within East Asia and ameliorated inequality amongst the upper-middle-income countries, but also mitigated global inequality to a large extent.

The findings have policy implications on the pre/post COVID times. Developing countries will be facing more challenges in tackling the pandemic due to lack of resources, thus our estimation results about North–South inequality will be even more severe in the aftermath of the pandemic. It is essential to assist poorer countries with more public goods, i.e., vaccines, to help low-income communities and those with poor sanitary environments, older people and those with a low awareness about the pandemic, so that more pertinent suggestions can be made for protecting the health of these subpopulations in the global south countries who are mostly affected by the pandemic.

## Data Availability Statement

Publicly available datasets were analyzed in this study. This data can be found here: World Bank Database: https://databank.worldbank.org/source/world-development-indicators.

## Author Contributions

NM: formal analysis and writing – original draft. TC: data curation, visualization, and funding acquisition. JL: conceptualization and writing – review and editing. All authors contributed to the article and approved the submitted version.

## Conflict of Interest

The authors declare that the research was conducted in the absence of any commercial or financial relationships that could be construed as a potential conflict of interest.

## Publisher’s Note

All claims expressed in this article are solely those of the authors and do not necessarily represent those of their affiliated organizations, or those of the publisher, the editors and the reviewers. Any product that may be evaluated in this article, or claim that may be made by its manufacturer, is not guaranteed or endorsed by the publisher.

## Footnotes

1
https://data.worldbank.org/country
